# Potential of known and short prokaryotic protein motifs as a basis for novel peptide-based antibacterial therapeutics: a computational survey

**DOI:** 10.3389/fmicb.2014.00004

**Published:** 2014-01-21

**Authors:** Heini Ruhanen, Daniel Hurley, Ambarnil Ghosh, Kevin T. O'Brien, Catrióna R. Johnston, Denis C. Shields

**Affiliations:** ^1^Complex and Adaptive Systems Laboratory, University College DublinDublin, Ireland; ^2^Conway Institute of Biomolecular and Biomedical Science, University College DublinDublin, Ireland; ^3^School of Medicine and Medical Science, University College DublinDublin, Ireland; ^4^Crystallography and Molecular Biology Department, Saha Institute of Nuclear PhysicsKolkata, India; ^5^Translational Research Institute, WoolloongabbaQLD, Australia

**Keywords:** short linear motifs (SLiMs), virulence factor, motif mimicry, antibacterial, bioinformatics, pathogen

## Abstract

Short linear motifs (SLiMs) are functional stretches of protein sequence that are of crucial importance for numerous biological processes by mediating protein–protein interactions. These motifs often comprise peptides of less than 10 amino acids that modulate protein–protein interactions. While well-characterized in eukaryotic intracellular signaling, their role in prokaryotic signaling is less well-understood. We surveyed the distribution of known motifs in prokaryotic extracellular and virulence proteins across a range of bacterial species and conducted searches for novel motifs in virulence proteins. Many known motifs in virulence effector proteins mimic eukaryotic motifs and enable the pathogen to control the intracellular processes of their hosts. Novel motifs were detected by finding those that had evolved independently in three or more unrelated virulence proteins. The search returned several significantly over-represented linear motifs of which some were known motifs and others are novel candidates with potential roles in bacterial pathogenesis. A putative C-terminal G[AG].$ motif found in type IV secretion system proteins was among the most significant detected. A KK$ motif that has been previously identified in a plasminogen-binding protein, was demonstrated to be enriched across a number of adhesion and lipoproteins. While there is some potential to develop peptide drugs against bacterial infection based on bacterial peptides that mimic host components, this could have unwanted effects on host signaling. Thus, novel SLiMs in virulence factors that do not mimic host components but are crucial for bacterial pathogenesis, such as the type IV secretion system, may be more useful to develop as leads for anti-microbial peptides or drugs.

## Introduction

Short linear motifs (SLiMs) are functional microdomains in proteins that play a critical role in many distinct biological processes such as cell signaling and regulation, post-translational modifications, proteolytic cleavage, and protein trafficking (Davey et al., 2011b; Mooney et al., 2012). These motifs are typically found in eukaryotic disordered protein regions and vary in size from 3 to 12 amino acids (Fuxreiter et al., 2007). In general, SLiMs have less than five defined amino acid positions and frequently these positions have some degree of flexibility in amino acid composition. Their shortness makes them evolutionarily plastic, allowing them to evolve convergently in unrelated proteins. This can allow proteins to rapidly acquire new protein interaction functions (Neduva and Russell, 2005; Diella et al., 2008; Davey et al., 2010, 2012b). Their short length also presents a challenge for SLiM discovery both experimentally and computationally, since there may be many false positive findings using both methods.

The presence of SLiMs in eukaryotes and viruses has been well-established. Several pioneering viral studies were crucial for the original characterization of SLiMs (Davey et al., 2011b). Viruses use SLiMs as a principal mechanism of hijacking cells by binding to host proteins and recruiting them to process viral proteins. A viral genome can contain various short motifs, many of which are necessary for the viral life cycle, providing a plethora of ways for the virus to take over the molecular machinery of the host cell (Kadaveru et al., 2008; Davey et al., 2011b). Like viruses, pathogenic bacteria are extremely proficient in intercepting host cell functions and in many cases it is still poorly understood how bacteria carry out the manipulation of the host cells. SLiMs have been documented in a number of cases to play a role in bacterial pathogenicity. However, bacterial linear motifs are not as well-characterized as in eukaryotes.

Most of the known instances of bacterial motifs are involved in pathogenicity including signals in effector proteins or host motif mimicry (Cornelis and Van Gijsegem, 2000; Alto et al., 2006). The tripeptide RGD motif is a known host extracellular matrix adhesion factor that is also used by bacteria to attach onto host cells (Tegtmeyer et al., 2010; Zimmermann et al., 2010; Zhang et al., 2012). RGD based anticancer and antithrombotic drugs are currently being developed but their direct impact on limiting bacterial adhesion and infectivity has not been investigated. A second example of a bacterial motif is the EPIYA motif found in several bacterial type III or IV secretion system effector proteins, which mimics SH2 binding peptides of the host (Hayashi et al., 2013). A third example of a bacterial motif has evolved to antagonize host proteins, but does this using a motif for which there is no eukaryotic equivalent. This W… E motif (where “.” indicates any amino acid) in bacterial effector proteins has been proposed to mimic host G-proteins (Alto et al., 2006; Jackson et al., 2008; Ham et al., 2009). Other motifs found in prokaryotes which are not simply mimicking known eukaryotic motifs play roles in transport, modification and proteolysis of the bacterial proteins (Table 1).

**Table 1:** **Examples of known instances of Short Linear Motifs in bacterial virulence factors**.

**Virulence factor**	**Motif**	**Function**	**References**
**ADHERENCE**			
CagL, Mce	RGD^*^, NGR	An integrin binding cell adhesion motif	Conradi et al., 2012a,b; Zhang et al., 2012
CagL	**FEANE**	Participates in integrin binding	Conradi et al., 2012b
YadA	**[SG][VI][AS][IVT]G..S**	Repeated collagen binding motif	Tahir et al., 2000
InlJ, InlA, nanA, CspA	**LP.TG**	Cell wall anchor motif	Harris et al., 2003; Sabet et al., 2005; Banerjee et al., 2010
Eno	[LF]Y[DNK]… [KG][KV]Y[VD]^*^	Plasminogen binding motif	Bergmann et al., 2003; Nogueira et al., 2012
CBPA-G, LytA-C	**W[WFY][FY]….G.M**	Repeated cholin (cell wall) binding motif	Garau et al., 2005
SpsA	**YRNYPT**	Host secretory immunglobulin A (SlgA) and secretory component (SC) binding motif	Hammerschmidt et al., 2000
**EFFECTOR**			
SipA, SopA	DEVD^*^, [DSTE][^∧^P][^∧^DEWHFYC]D[GSAN]	Caspase 3 cleavage site motif	Srikanth et al., 2010; Dinkel et al., 2012
AvrPto, HopF2, AvrB, AvrRpm1	MG..C^*^,G… [STC], ^∧^M{0, 1}(G)[^∧^EDRKHPFYW]..[STAGCN][^∧^P]	*N*-myristoylation/*S*-palmitoylation motif	Shan et al., 2000; Robert-Seilaniantz et al., 2006; Dinkel et al., 2012; Hicks and Galan, 2013
WtsE, AvrE1, IpgB2, IpgB1, Map, EspM, EspT, SifA, SifB	W… E^*^	Host Rho GTPase activation/modulation motif	Alto et al., 2006; Ham et al., 2009
WtsE,	[LR][KQVS][KQLR][EST][GQR][FLKS][EGPK] [MLVAS][KNAL][SGIE]^*^	Putative endoplasmic reticulum membrane retention/retrieval motif	Ham et al., 2006, 2009
SifA, AnkB	CLCCFL^*^, (C)[^∧^DENQ][LIVM].$	CAAX box, putative prenylation motif (addition of farnesyl or geranylgeranyl group)	Boucrot et al., 2003; Hicks et al., 2011; Dinkel et al., 2012
YopE, SptP, ExoS	G.LR… T(YopE^*^)	Arginine finger motif, essential for Rho GAP function of virulence factors	Black and Bliska, 2000; Wurtele et al., 2001
PopB, PopP2, AvrBs3	[^∧^DE]((K[RK])|(RK))[KRP][KR][^∧^DE], [KR][KR].{7,15}[^∧^DE]((K[KR])|(RK))(([^∧^DE][KR]) |(KR][^∧^DE]))[^∧^DE], [^∧^DE]((K[RK])|(RK))(([^∧^DE][KR])|([KR][^∧^DE]))(([PKR]) |([^∧^DE][DE])), (([PKR].{0,1}[^∧^DE])|([PKR]))((K[RK])|(RK)) (([^∧^DE][KR])|([KR][^∧^DE]))[^∧^DE]	Nuclear localization signal (NLS) motifs	Szurek et al., 2002; Deslandes et al., 2003; Dean, 2011; Dinkel et al., 2012
CagA, Tarp, AnkA, LspA	E[PNS][IV]Y[AEG]	Membrane targeting/phosphorylation motif	Higashi et al., 2005; Suzuki et al., 2009; Hayashi et al., 2013
SspH2, SseI	….GSGC….., G(C)M[GS][CL][KP]C, ^∧^M{0,1}G(C)..S[AKS]	*S*-palmitoylation motif	Hicks et al., 2011; Dinkel et al., 2012
ExoS, SopE	[FIV]..[FIV].[FIV]..[NC].[FIV]	Membrane localization motif (targets ExoS to the Golgi-endoplasmic reticulum)	Zhang and Barbieri, 2005
SopD2, SifA, SseJ, SspH2	**WEK[IM]..FF**	Translocation/late endocytic compartments targeting motif	Brown et al., 2006
ExoU	**KAWRN**	Plasma membrane localization/ubiquitinylation motif	Rabin and Hauser, 2005; Stirling et al., 2006
SopE, BopE	**GAG[AT]**	Catalytic loop motif essential for guanine nucleotide exchange	Schlumberger et al., 2003
AvrPphB	**GDK**	Autoproteolytic cleavage motif	Dowen et al., 2009
SopA, IpaH, SspH1	L….TC, C.D	E3 ubiquitin ligase motif	Zhang et al., 2006; Rohde et al., 2007
VirF	LP… … ….L	F-box domain motif, mediates protein–protein interactions	Tzfira et al., 2004
VopL, VopF	[^∧^R]..((.[ILMVF])|([ILMVF].))[^∧^P][^∧^P][ILVM]. {4,7}L(([KR].)|(NK))[VATIGS], [R]..[ILVMF][ILMVF][^∧^P][ILVM].{4,7}L(([KR].) |(NK))[VATI]	WH2-domain motif	Liverman et al., 2007; Dinkel et al., 2012
SpvC, OspF, VirA	[KR]{0,2}[KR].{0,2}[KR].{2,4}[ILVM].[ILVF]	D motif, Docking motif required for specific binding to MAPKs	Zhu et al., 2007; Dinkel et al., 2012
IpaA	L..AA..VA..V..LI..A.	Vinculin binding domain motif	Hamiaux et al., 2006
ExoS, ExoT	**LLDALDLA**	FAS (14-3-3 protein) binding motif, mediates activation of the ADPRT domain	Sun et al., 2004; Dean, 2011
EspF	[RKY]..P..P, P..P.[KR],…[PV]..P, KP..[QK]…	SH3 binding motif	Alto et al., 2007; Dinkel et al., 2012
Map, NleH1, EspI (NleA)	…[ST].[ACVILF]$, … [VLIFY].[ACVILF]$,…[DE].[ACVILF]$ (EspI^*^)	C-terminal PDZ1 binding motif	Lee et al., 2008; Martinez et al., 2010; Dinkel et al., 2012
**TOXIN**			
Listeriolysin O (LLO)	PPASP^*^	PEST-motif, involved in phagosomal escape of bacteria in infected cells	Lety et al., 2001
**OTHER**			
VirD4, VirB11, VirB4, SecA	G….GK[TS]^*^	Walker A motif, nucleotide-binding motif	Sato et al., 1996; Atmakuri et al., 2004
SecA	[RK]….G….L[VILFWYMC]{4,4}D	Walker B, nucleotide binding motif	Sato et al., 1996
MsbA, PiaA, PiuA	LSGGQ (PiaA^*^, PiuA^*^)	ABC-motif, ATP binding cassette transporter motif	Garmory and Titball, 2004; Buchaklian and Klug, 2006
EsxA, EsxB, esat6	**W.G**	W.G motif helps to create a shallow cleft structure and may represent a peptide recognition feature by which cargo proteins are acquired for transport	Burts et al., 2005; Sundaramoorthy et al., 2008

* *Proven role in virulence. Bold, a non-eukaryotic motif*.

“∧” start of the protein or if in the middle of the motif sequence states which amino acids are excluded in the position,

*“$” end of the protein, “.” any amino acid, {} defines the length of a range in the motif sequence, [] defines which amino acids can occur at a given motif position, () marks positions of specific interest e.g., covalent modification or is used to group parts of the expression. Motif table modified from Dean (2011), additional motifs added from the literature*.

Since SLiMs are used in a plethora of cellular processes in eukaryotes and are utilized by both pathogenic bacteria and viruses, discovering and characterizing new linear motifs is of great importance. As well as shedding light on the mechanisms of fundamental cellular processes they also hold promise as future therapeutic targets. There is an urgent need for new classes of antimicrobial therapeutics that are effective against multidrug resistant bacteria. Conventional antibiotics are becoming increasingly ineffective against pathogenic bacteria, such as methicillin resistant *Staphylococcus aureus* (MRSA) which presents a severe threat to public health.

We were interested in whether SLiMs may be valuable when developing new antimicrobial peptides or drugs. Compared with recombinant proteins, the smaller size of peptides makes them easier to manufacture and deliver. The use of chemically synthesized peptides in pharmacological and clinical applications is relatively limited by their low systemic stability and high clearance, poor membrane permeability, negligible activity when administered orally and their high cost of manufacture in comparison to small chemical compounds. However, to date more than 100 peptide-based drugs have already reached the market and of these, the majority are at the smaller end of the size spectrum at 8–10 amino acids (Craik et al., 2013).

Here, we conducted a study to discover SLiMs computationally in bacterial virulence factor datasets. We surveyed the distribution of these novel motifs, and compared their distribution with that of known motifs observed in prokaryotic proteins. The list of motifs given here represents a useful resource for experimental scientists interested in targeting SLiMs that may be important for the pathogenicity of bacteria.

## Materials and methods

We utilized data from a virulence factor database MvirDB (Lawrence Livermore National Laboratory), which integrates DNA and protein sequence information from Tox-Prot, SCORPION, the PRINTS database of virulence factors, VFDB, TVFac, Islander, ARGO, CONUS, KNOTTIN, a subset of VIDA and sequences derived by means of literature searches (Zhou et al., 2007). MvirDB can be accessed at http://mvirdb.llnl.gov. The MvirDB browser tool was used to search the database to retrieve virulence factors by functional categories (Table 2) and to download sequences of interest. Protein sequence identifiers for the downloaded sequences for each functional category are available in Table S1.

**Table 2:** **Functional search terms used to retrieve and download protein sequences from virulence factor database MvirDBbrowser tool**.

**Virulence protein group**	**Number of sequences**	**Number of unrelated proteins**
Adherence	749	181
Capsule	332	57
Chemotaxis	192	18
Effector	111	27
Endotoxin	66	27
Enzyme	647	121
Exotoxin	92	12
Lipoprotein	463	70
Motility	86	23
Siderophore	150	43
Type III secretion system	571	75
Type IV secretion system	181	38

The recovered protein sequences in each functional category thought to be associated with pathogenicity were searched for SLiMs using SLiMFinder (Davey et al., 2010) both locally, and on a webserver that is available at http://bioware.ucd.ie. The default settings provided in SLiMFinder without any extra masking were used in the analysis. This method finds sets of three or more unrelated proteins in a dataset of proteins that share a motif. Chemotaxis and enzyme protein sequence datasets were filtered to contain only sequences longer than 20 amino acids and lipoprotein and Exotoxin datasets sequences longer than 40 amino acids prior to the analysis.

The motifs identified by the SLiMFinder analysis were further examined for similarity to known SLiMs from literature motifs using CompariMotif, which takes two lists of protein motifs and compares them to each other, identifying and scoring similarities between short motifs in the sets (Edwards et al., 2008).

Motifs were visualized using the MEME Suite (Bailey et al., 2009), by taking a stretch of 10 amino acid residues containing the motif of interest from each protein sequence where the motif was found. MEME represents motifs as position dependent letter probability matrices which describe the probability of each possible letter at each position in the pattern. These are displayed as “sequence LOGOS,” containing stacks of letters at each position in the motif. The total height of the stack is the “information content” of that position in the motif in bits. The height of the individual letters in a stack is the probability of the letter at that position multiplied by the total information content of the stack.

Datasets comprised of protein sequences obtained from UniProtKB that are predicted to be effector proteins from a selection of 60 organisms represented in the MvirDB were used to assess the distribution of prokaryotic protein motifs. The presence of both known and novel motifs in these datasets was investigated using the predictive computational tool SLiMSearch which can be used to determine the occurrences of predefined motifs in protein sequences (Davey et al., 2011a). Heat maps were generated to visualize the incidences of motifs in the protein datasets where the frequency of the heat map represents the logarithm of the normalized N_UPC (Number of incidences of a motif in an Unrelated Protein Cluster) value returned in the SLiMSearch results. The N_UPC for an individual motif in a specific organism was normalized by dividing the value by the total amount of UPCs (Unrelated Protein Clusters) in the specific organism and the average N_UPCs of a motif across all 60 organisms. For motifs where there were no incidences in a specific organism the frequency was set to an arbitrary value lower than the minimum actual observed value.

The organisms in Figures 2, 3 which cover the motif sequences were presented in a phylogenetic tree (Figure 4). The Taxonomic IDs for all the organisms are used as input in NCBI's Taxonomy Common Tree tool (http://www.ncbi.nlm.nih.gov/Taxonomy/CommonTree/wwwcmt.cgi). The “phenogram” taxonomic tree (^*^.phy format) obtained from the NCBI server was fed into Drawgram tree drawing program of Phylip package (version 3.695). Branches were colored according to the following scheme: Purple, High GC Gram+ bacteria; Blue, Firmicutes; Yellow, a-proteobacteria; Light Brown, b-proteobacteria; Dark Brown, e-proteobacteria; Green, g-proteobacteria (non-enterobacteria); Red, g-proteobacteria (enterobacteria); Black, others (CFB).

## Results

Our objective was to discover novel SLiMs in non-homologous bacterial proteins with similar roles in virulence that may have functional importance in pathogenesis, and thus have potential to be developed into antimicrobial peptides or drugs. Our analysis returned both previously characterized and novel motifs in several different functional categories indicating the suitability of SLiMFinder for the analysis of bacterial sequence data as well as eukaryotic data. We focused on 12 groups of bacterial proteins with predefined roles in pathogenicity (Table 2). SLiMFinder identified numerous motifs among these proteins. Table 3 lists those with a *p*-value (Sig) less than 0.05. Bonferroni correction for significance with 12 search datasets would suggest that motifs with a Sig value of less than 0.004 are significant. Since pathogenesis proteins from bacteria often interact with host protein components, we examined whether any of the identified motifs showed similarity to known eukaryotic linear motifs, using the Comparimotif tool. However, we did not find any convincing similarities, in spite of the known occurrence of eukaryotic motifs in bacterial effector proteins. We also investigated if any of the motifs were known prokaryotic motifs identified in the literature.

**Table 3:** **Significant motifs returned by SLiMFinder in each dataset (where probability <0.05)**.

**Virulence protein group**	**Number of sequences**	**Motif pattern**	**Motif name**	**Information content**	**Occurrences**	**Unrelated proteins**	**Probability (Sig value)**	**References**
**(A) PREVIOUSLY DESCRIBED MOTIFS**								
Adherence	749	^∧^.K.{0,2}K	Signal peptide motif	3	151	47	0.00E+00	Juncker et al., 2003; Bagos et al., 2008
		^∧^.{1,2}K.{1,2}L	Signal peptide motif	3	102	35	1.15E–10	Juncker et al., 2003; Bagos et al., 2008
		^∧^.{1,2}K.{0,2}I	Signal peptide motif	3	91	34	1.41E–08	Juncker et al., 2003; Bagos et al., 2008
		^∧^.K.[IL]	Signal peptide motif	2.77	58	21	5.80E–07	Juncker et al., 2003; Bagos et al., 2008
		^∧^.K..S	Signal peptide motif	3	24	12	0.002	Juncker et al., 2003; Bagos et al., 2008
		KK$	C-terminal KK	3	23	11	0.034	Bergmann et al., 2003; Itzek et al., 2010
Capsule	332	^∧^.{1,2}K.{0,1}I	Signal peptide motif	3	55	18	6.96E–06	Juncker et al., 2003; Bagos et al., 2008
		^∧^.{1,2}K.{0,1}I..V	Signal peptide motif	4	16	7	0.002	Juncker et al., 2003; Bagos et al., 2008
		^∧^.{1,2}K.{0,2}I	Signal peptide motif	3	59	18	0.003	Juncker et al., 2003; Bagos et al., 2008
		^∧^.K.[ILV]	Signal peptide motif	2.63	39	13	0.003	Juncker et al., 2003; Bagos et al., 2008
		^∧^.{1,2}K.{0,2}K	Signal peptide motif	3	52	17	0.006	Juncker et al., 2003; Bagos et al., 2008
Chemotaxis	192	–		–	–	–	–	–
Effector	111	–		–	–	–	–	–
Endotoxin	66	–		–	–	–	–	–
Enzyme	647	^∧^.{1,2}KK	Signal peptide motif	3	27	14	9.52E–05	Juncker et al., 2003; Bagos et al., 2008
		^∧^..K.{0,2}I	Signal peptide motif	3	30	13	0.042	Juncker et al., 2003; Bagos et al., 2008
Exotoxin	92	–		–	–	–	–	–
Lipoprotein	463	L.[AG]C[AGS]	Lipobox	3.4	78	30	0.00E+00	Braun and Rehn, 1969; Babu et al., 2006
		[FLV].L.[AG]C	Lipobox	3.4	136	24	0.00E+00	Braun and Rehn, 1969; Babu et al., 2006
		[ILV].[AGS]C	Lipobox	2.27	370	53	0.00E+00	Braun and Rehn, 1969; Babu et al., 2006
		[AGS]C[AGS]	Lipobox	2.27	285	50	7.22E–15	Braun and Rehn, 1969; Babu et al., 2006
		^∧^.{1,2}K.{0,2}K	Signal peptide motif	3	109	27	1.27E–09	Juncker et al., 2003; Bagos et al., 2008
		L.{1,2}GC.{0,1}A	Lipobox	4	41	15	1.65E–09	Braun and Rehn, 1969; Babu et al., 2006
		A.{0,2}L..C.{0,2}S	Lipobox	4	68	19	3.02E–09	Braun and Rehn, 1969; Babu et al., 2006
		^∧^.K..[ILV]	Signal peptide motif	2.63	66	19	4.36E–09	Juncker et al., 2003; Bagos et al., 2008
		^∧^..K..[FLV]	Signal peptide motif	2.63	65	17	1.07E–07	Juncker et al., 2003; Bagos et al., 2008
		[ILV]..C.[AGS]	Lipobox	2.27	217	36	4.11E–06	Braun and Rehn, 1969; Babu et al., 2006
		KK$	C-terminal KK	3	22	9	0.016	Bergmann et al., 2003; Itzek et al., 2010
Motility	86	–		–	–	–	–	–
Siderophore	150	^∧^.[KR]I	Signal peptide motif	2.77	12	7	0.024	Juncker et al., 2003; Bagos et al., 2008
Type III secretion	571	–		–	–	–	–	–
Type IV secretion	181	^∧^.K[KR]	Signal peptide motif	2.77	29	9	0.003	Juncker et al., 2003; Bagos et al., 2008
		^∧^.K..[FIL]	Signal peptide motif	2.63	27	10	0.025	Juncker et al., 2003; Bagos et al., 2008
**(B) NOVEL MOTIFS**								
Adherence	749	LP.G.Y		4	37	12	0.012	
Capsule	332	G.S..M.L		4	15	7	0.029	
Chemotaxis	192	E..Q.I[AG].I		4.77	22	5	0.004	
		E..Q.[IV]..I		3.77	24	7	0.02	
Effector	111	^∧^..I.{0,1}N		3	21	6	0.012	
		[LV].PY		2.77	46	11	0.042	
		^∧^..I[ST]		2.77	17	6	0.049	
Endotoxin	66	–		–	–	–	–	
Enzyme	647	A.I.P.VL		5	14	7	0.019	
		VSIL.S		5	11	7	0.049	
Exotoxin	92	–		–	–	–	–	
Lipoprotein	463	ML..C		3	14	7	0.017	
Motility	86	–		–	–	–	–	
Siderophore	150	I.K..G		3	28	17	0.044	
		GYP..TP		5	5	4	*0.052*	
Type III secretion	571	–		–	–	–	–	
Type IV secretion	181	G[AG].$		2.77	19	9	2.64E–04	

*Where very similar motifs are returned for a protein group, only a representative motif is shown*.

Information content (Edwards et al., 2007),

“^∧^” *start of the protein, “$” end of the protein, “.” any amino acid, {} defines the range of a repeat in the motif sequence, [] defines which amino acids can occur at a given motif position*.

*Italic font is used when Probability (Sig-value) is higher than the 0.05 confidence level*.

### Known motifs

Three of the motifs highlighted by SLiMFinder were previously known bacterial motifs. The most significant of these was the well-characterized prokaryotic N-terminal lipid modification [LVI][ASTVI][GAS]C motif that has been previously shown to be essential for the anchoring of bacterial proteins to the membrane surface (Braun and Rehn, 1969; Babu et al., 2006). The square brackets enclose alternative amino acids which are possible at that position in the motif. This motif is present in a wide range of proteins across Gram-positive and Gram-negative bacteria and is a clear example of a motif that has convergently evolved in many unrelated proteins. It was found in numerous configurations in the lipoprotein dataset of which seven are listed in Table 3. This “lipobox” motif sequence is located at the C-terminal end of the signal peptide and the lipid-modifiable cysteine (+1 position) is invariant (Juncker et al., 2003). Lipid modification of this cysteine residue (*N*-acyl-*S*-diacylglyceryl-Cys) has been found to be an essential, ubiquitous, and unique bacterial post-translational modification. Such a modification allows anchoring of even highly hydrophilic proteins to the membrane surface leaving the rest of the protein to carry out a variety of relevant functions in the aqueous or aqueous-membrane interface (Juncker et al., 2003; Babu et al., 2006). Bacterial lipoproteins affect a wide range of mechanisms in virulence. They have been shown to play key roles in adhesion to host cells and in translocation of virulence factors into host cells (Kovacs-Simon et al., 2011). Furthermore, they are potent inducers of host inflammatory responses.

**Figure 1:**
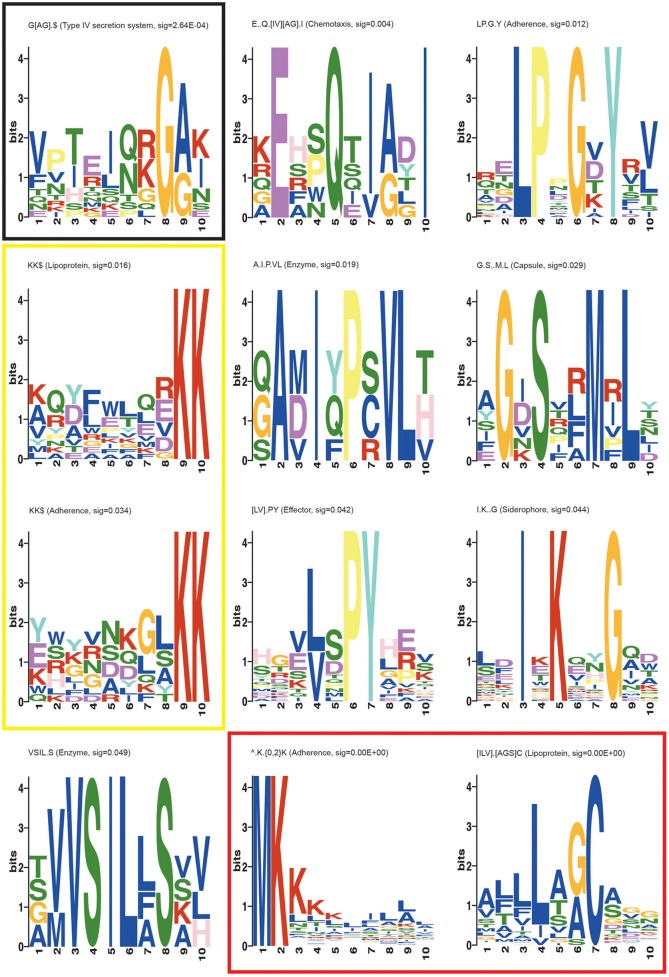
**MEME suite motif logos of the novel and known motifs returned in the SLiMFinder analysis**. Each position in the motif is represented as a stack of letters. The total height of the stack is the “information content” of that position in the motif in bits. The height of the individual letters in a stack is the probability of the letter at that position multiplied by the total information content of the stack. Black box: the most significant novel motif G[AG].$, Yellow box: KK$ motifs found in Adherence and Lipoprotein datasets, Red box: Known bacterial motifs ^∧^.K.{0,2}K and [ILV].[AGS]C.

The second known motif identified was an N-terminal ^∧^MK.{0,2}K motif present in several search categories in varying configurations (Table 3, Adherence, Capsule, Enzyme, Lipoprotein, Siderophore, and Type IV secretion system). This motif representation indicates that the second K (lysine) may lay 0, 1, or 2 residues after the K that follows the initiator methionine. The “^∧^” symbol indicates the start of the protein, which is treated as a distinct character in motif discovery. SLiMFinder omits the M from the returned motif resulting in ^∧^.K.{0,2}K representation, since initiator methionines were deliberately masked out to avoid returning motifs reliant simply on the strong enrichment of M at the start of proteins. The ^∧^MK.{0,2}K motif is commonly found in bacterial signal peptides both in proteins that are targeted to the membrane and in secreted proteins (Juncker et al., 2003; Bagos et al., 2008). Both of the known motifs are presented as regular expressions in Figure 1, which provides some information on additional contextual preferences beyond the simple motif description. Signal peptides in bacteria are mainly divided into the secretory signal peptides that are cleaved by Signal Peptidase I and those cleaved by Signal Peptidase II which characterize the membrane-bound lipoproteins (Juncker et al., 2003; Bagos et al., 2008). The signal peptides in both classes of proteins in Gram-positive and Gram-negative bacteria are quite similar, sharing the N-terminal region which is characterized by presence of the positive amino acids at the start of the protein, as well as the preference for hydrophobic residues further along the signal peptide.

The third previously characterized bacterial motif returned in our analysis is the C-terminal KK$ motif (where $ indicates the end of the protein, and is treated as a distinct character in motif discovery) found in adherence and lipoprotein datasets (Table 3; Figure 1). This motif has been shown to play a role in plasminogen binding in *S. pyogenes* and *S. pneumoniae* α-enolase (Bergmann et al., 2003; Derbise et al., 2004; Itzek et al., 2010). Binding of plasminogen by α-enolase and its subsequent activation has been demonstrated to promote invasion of pathogenic bacteria and therefore represents an important determinant of virulence in invasive infection (Bergmann et al., 2003). Moreover, KK motifs close to the C-terminus are present in a family of *Shigella flexneri* glucosyl transferases (Gtr) that are integral membrane proteins embedded within the cytoplasmic membrane. These glucosyl transferases contribute to the altering of the structure of the bacterial surface lipopolysaccharide (LPS) O-antigen along with O-acetyltransferase (Lehane et al., 2005; Ramiscal et al., 2010). The KK motif has been shown to be essential for the activity of Gtrs. However, Ramiscal et al. showed that the KK motif in a recently identified GtrIc is not critical for its activity (Ramiscal et al., 2010). We hypothesize that the KK$ motif instances identified here in diverse proteins may play an adhesive role similar to the plasminogen binding instances in α-enolase. We note that plants have a KK$ variant (Gidda et al., 2009) of a known eukaryotic cytoplasmically exposed endoplasmic reticulum (ER) localization motif KKxx$ found in mammals, yeast and plants (Nilsson et al., 1989; Jackson et al., 1990; Contreras et al., 2004). It is therefore conceivable that the bacterial KK$ motif could in some proteins direct invading proteins to certain parts of the eukaryotic host cell. However, we do not think this is very plausible, since the enrichment of KK$ motifs spans many known bacterial lipoproteins (Table 3) which seem unlikely to migrate to this host cell location.

### Novel motifs

**Figure 2:**
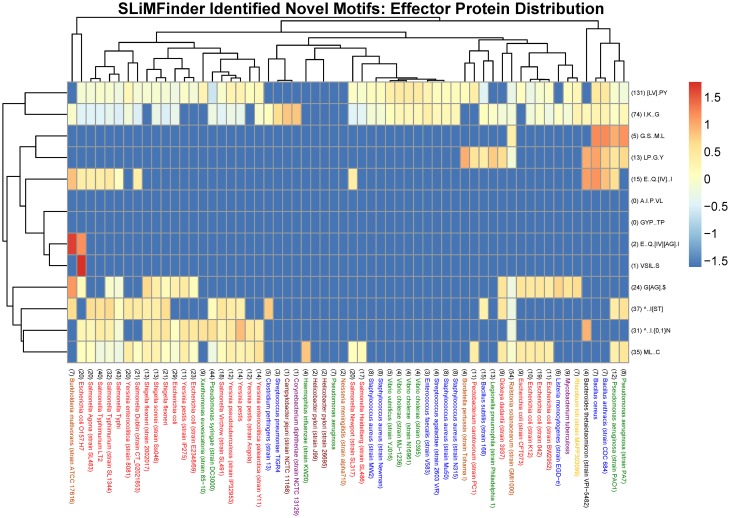
**Heat map visualization of the distribution of novel SliMFinder identified motifs amongst effector proteins from a selection of 60 organisms represented in the MvirDB**. Columns: The bacterial species name with the total number of UPCs indicated in brackets at the start of the description. Purple, High GC Gram+ bacteria; Blue, Firmicutes; Yellow, a-proteobacteria; Light Brown, b-proteobacteria; Dark Brown, e-proteobacteria; Green, g-proteobacteria (non-enterobacteria); Red, g-proteobacteria (enterobacteria); Black, others (CFB). Rows: The motif regular expression with the total number of incidences in UPCs across all 60 organisms indicated in brackets at the start of the description. Color scale: The logarithm of the normalized N_UPC returned from the SLiMSearch results.

The most significant novel motif (*p*-value 0.0003) discovered is a C-terminal G[AG].$ motif in the type IV secretion system dataset. The full list of unrelated proteins containing the G[AG].$ motif is represented in Table 4. The MEME regular expression pattern of the motif in these proteins is described in Figure 1. Four of the nine unrelated proteins containing this motif appear to be identified equivalents of the type IV secretion system components in the well-studied *Agrobacterium tumefaciens*: VirB4, VirB8, VirB11, and VirB7 [TrwH has 59% identity with VirB7 family (Patey et al., 2006)]. VirB4 and VirB11 are known energetic components of the type IV secretion system in *A. tumefaciens*. Both of these proteins are membrane associated NTPases on the inner membrane (Tegtmeyer et al., 2011). VirB8 on the other hand, is an essential inner membrane component of type IV secretion systems that is believed to form a homodimer and has been shown to be of importance for complex stability in *A. tumefaciens* (Sivanesan and Baron, 2011). The VirB7 is an outer membrane lipoprotein that localizes exocellularly and associates with the type IV secretion system pilus. Both VirB7 lipid modification and disulfide cross-linking have been shown to be important for pilus assembly (Sagulenko et al., 2001). The *Helicobacter pylori* protein Cag7 that is among the proteins containing the C-terminal G[AG].$ motif has previously been proposed to be a transmembrane protein that is associated with the pilus (Rohde et al., 2003; Tegtmeyer et al., 2011). At least five of the nine unrelated proteins containing the G[AG].$ motif seem to be associated with the bacterial membranes and it is thus possible that this motif would be involved in the targeting and/or attachment of these proteins into the bacterial membranes. However, since the motif has been specifically identified within type IV secretion proteins, it is more likely that the motif facilitates interaction with a component of the type IV secretion system itself. We inspected the distribution of the motif across effector proteins (Figure 2) and noted that there are typically one or none per species, suggesting that the motif is not itself enriched strongly among effector proteins themselves.

**Table 4:** **List of proteins containing G[AG].$ and KK$ motifs**.

**Pattern**	**Protein group**	***P*-value (Sig)**	**Match**	**No. unrelated proteins**	**Description**
G[AG].$	Type IV secretion	2.64E–04	GGN	9	virB11 protein homolog|9992|YP_034060|49476019|8040| [*Bartonella henselae* str. Houston-1]
			GAK		virB4|10558|NP_863348|32469876|8343|VirB4 [*Campylobacter jejuni* subsp. *jejuni* 81-176]
			GAK		virB8|10560|NP_863298|32469826|8344|VirB8 [*Campylobacter jejuni* subsp. *jejuni* 81-176]
			GAE		cag pathogenicity island protein (cag11)|10866|NP_207327|15645157|8497| [*Helicobacter pylori* 26695]
			GGK		trwF protein|9938|YP_034270|49476229|8013|[*Bartonella henselae* str. Houston-1]
			GAI		trwH2 hypothetical protein BH15720|9944|YP_034268|49476227|8016 [*Bartonella henselae* str. Houston-1]
			GAS		cag pathogenicity island protein (cag25)|10894|NP_207342|15645172|8511| [*Helicobacter pylori* 26695]
			GGN		Putative type IV secretion system protein|41299|NP_790379|NP_790379.1|18355| [*Pseudomonas syringae* pv. tomato str. DC3000]
			GAI		trwH1 hypothetical protein BH15690|9942|YP_034265|49476224|8015| [*Bartonella henselae* str. Houston-1]
			GGN		cag pathogenicity island protein (cag7)|10904|NP_207323|15645153|8516| [*Helicobacter pylori* 26695]
KK$	Adherence	0.034	KK	11	hmw2C putative accessory processing protein [*Haemophilus influenzae*]|2847|AAA20526|482843|2554|
			KK		kpsC polysaccharide modification protein [*Campylobacter jejuni* subsp. *jejuni* NCTC 11168]|10454|NP_282555|15792732|8291|
			KK		ica operon transcriptional regulator [*Staphylococcus aureus* subsp. aureus MW2]|3137|NP_647402|21284314|2699|
			KK		pavA adherence and virulence protein A [*Streptococcus agalactiae* 2603V/R]|9719|NP_688199|22537348|7896|
			KK		Type 4 fimbrial biogenesis protein PilO [*Pseudomonas aeruginosa* PAO1]|8738|NP_253729|15600235|7426|
			KK		Type 4 fimbrial biogenesis protein PilN [*Pseudomonas aeruginosa* PAO1]|8740|NP_253730|15600236|7427|
			KK		Putative collagen binding protein [*Streptococcus pyogenes* MGAS315] SpyM3_0098|9709|NP_663902|21909634|7891|
			KK		oapA opacity associated protein [*Haemophilus influenzae* Rd KW20]|2865|NP_438494|16272282|2563|
			KK		neuC1 putative N-acetylglucosamine-6-phosphate 2-epimerase/N-acetylglucosamine-6-phosphatase [*Campylobacter jejuni* subsp. *jejuni* NCTC 11168]|10349|NP_282290|15792467|8218|
			KK		waaE D,D-heptose 1-phosphate adenosyltransferase/7-phosphate kinase [*Campylobacter jejuni* subsp. *jejuni* NCTC 11168]|10396|NP_282297|15792474|8262|
			KK		hmw1C putative accessory processing protein [*Haemophilus influenzae*]|2841|AAA20529|475773|2551|
			KK		cytotoxin [*Escherichia coli* O157:H7]|11176|AAC70163|3822209|8652|
KK$	Lipoprotein	0.016	KK	9	Multidrug resistance outer membrane efflux protein mdtP; Flags: Precursor|58083|Q8CVH8|24068| *Escherichia coli*
			KK		ylpB/yscJ needle complex inner membrane lipoprotein [*Yersinia pestis* CO92]|20866|NP_395193|16082747|13354|
			KK		Yop proteins translocation lipoprotein J OS = *Yersinia enterocolitica* GN = yscJ PE = 2 SV = 1|18600| AltName: Full = Lipoprotein ylpB; Flags: Precursor; |Q01251|11895|
			KK		Lipoprotein [*Salmonella enterica* subsp. *enterica* serovar Typhi str. CT18]|44580|NP_455466|NP_455466.1|19996|
			KK		LPP20 lipoprotein OS = *Helicobacter pylori* GN = lpp20 PE = 1 SV = 1|17433|P0A0V0|10728|
			KK		Outer membrane factor of efflux pump [*Escherichia coli* str. K-12 substr. MG1655]58055|NP_418504|NP_418504|24053|
			KK		Lipoprotein, putative [*Enterococcus faecalis* V583]9889|NP_816134|29376980|7982|
			KK		Iron transport lipoprotein SirF [*Staphylococcus aureus* subsp. *aureus* Mu50]|8986|NP_371657|15924123|7550|
			KK		Export protein prsA cytoplasmic membrane protein, protein folding|3480|GBAA2336 prsA-3 |GBAA2336|2870|
			KK		LPP20 lipoprotein OS = *Helicobacter pylori* J99 GN = lpp20 PE = 3 SV = 1|17432|P0A0V1|
			KK		Yop proteins translocation lipoprotein J OS = *Yersinia pseudotuberculosis* GN = yscJ PE = 2 SV = 1|18863| AltName: Lipoprotein ylpB |P69973|12158|
			KK		YaeC family lipoprotein [*Enterococcus faecalis* V583] [*Enterococcus faecalis* V583]|39473|NP_815743|NP_815743.1|17442|
			KK		Major outer membrane lipoprotein OS = *Yersinia pestis* GN = lpp PE = 3 SV = 1|24369| |Q8ZDZ6|15171|
			KK		Putative lipoprotein [*Salmonella enterica* subsp. *enterica* serovar Typhi str. Ty2]|44956|NP_805720|NP_805720.1|20184|

Other novel motifs discovered are summarized in Table 3 and in Figure 1. Their significance is in the range between that for the nominal significance level (*p* < 0.05) and the Bonferroni adjusted significance level (*p* < 0.004). While it is likely that a number of these motifs are genuine, a few may be false positives. The LP.G.Y motif found in the adherence dataset superficially resembles a Gram-positive bacteria cell wall anchoring LP.TG motif. Cleavage between the Thr and Gly by sortase or a related enzyme leads to covalent anchoring of the new C-terminal Thr to the cell wall (Navarre and Schneewind, 1994; Gaspar et al., 2005). Cell wall-anchored surface proteins of Gram-positive pathogens play important roles during the establishment of many infectious diseases. While it could be hypothesized that the LP.G.Y motif is similarly involved in the anchoring of bacterial proteins to the cell surface, there are two lines of evidence that argue against this. Firstly, there is no enrichment for T or similar amino acids between P and G in the instances of the motif returned (Figure 1). Secondly, this motif is present both in Gram-positive and Gram-negative bacterial proteins in our study. Accordingly, we consider LP.G.Y a potential novel motif involved in bacterial adhesion through an unidentified mechanism.

### Repeated motifs

While SLiMFinder looks for motifs which recur one or more times in a number of independent proteins, it is of biological interest when those motifs are themselves repeated within the proteins, for example, representing multiple adhesion sites. Accordingly, we investigated the frequency of repeats of the identified motifs. Duplicated motifs were found with between two and four copies in proteins. The lipoprotein lipid anchoring motif was found repeated three times in the protein HrpB3 of *Xanthomonas euvesicatoria* (instances LAGC, LALC, and LSAC). Among these motif instances, LAGC and LSAC are known lipid anchoring motifs (Klein et al., 2005; Konkel et al., 2010). The third instance may represent a true positive anchoring motif, a degenerate motif that is no longer functional or a false positive sequence that fulfills some other functional role in the protein. However, it is clear that the repetition of this well-known motif is in some cases biologically important for function. Thus, for novel motifs, repetition within as well as between proteins may be a potential further indication of important function. An example would be the threefold repetition of the “LP.G.Y” motif in the surface-anchored fimbrial subunit protein SpaG of *Corynebacterium diptherae*. This motif has a known structure in the collagen binding domain of *Staphylococcus aureus* (PDB entry 1D2P) (Deivanayagam et al., 2000). Collagen is itself a repetitive structure, occurring in many dense repeats in the host extracellular matrix. The repetition of this bacterial motif in this particular protein may indicate its potential role in making multiple contacts with collagen. However, other instances of the motif detected by SLiMFinder only occurred once in each protein, suggesting that a single copy may be sufficient.

### Distribution of short linear motifs across effector proteins of different species

We visualized the cross-species distribution of the SLiMFinder identified novel motifs (see Table 3B) among the annotated effector proteins of other species. The species were chosen to include those present in the MvirDB database that contributed motifs to the discovery, in order to display a varied set of species that could be visualized with ease. It is likely that they also exist in other organisms, although distinguishing true and false positives is not possible computationally. The visualization is normalized to correct for the fact that some species have very few proteins and that some motifs have very few instances. The total number of UPCs are indicated in brackets before each bacterial species as well as the total incidences of a motif in UPCs across all bacterial species indicated before each motif regular expression. The novel SLiMFinder identified effector protein motifs ^∧^..I.{0,1}N, [LV].PY and ^∧^..I[ST] are found among the effector proteins of many species, but are absent in those of many other species, including those with a reasonable number of annotated effector proteins (Figure 2).

**Figure 3:**
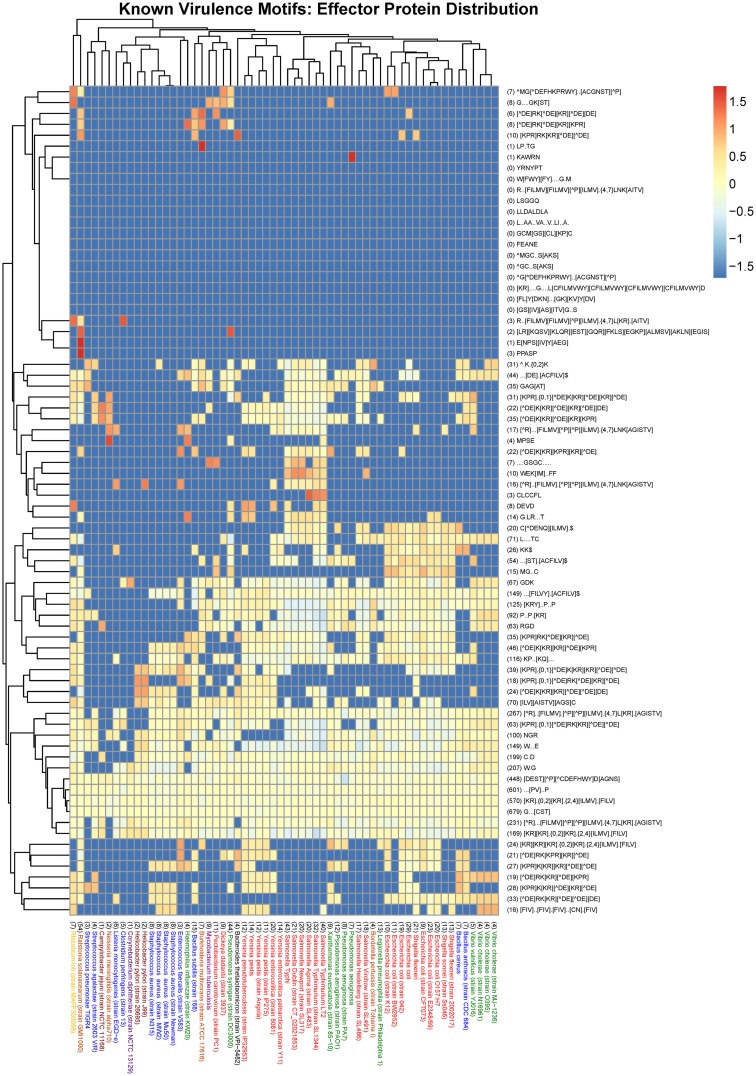
**Heat map visualization of the distribution of known virulence motifs amongst effector proteins from a selection of 60 organisms represented in the MvirDB**. Columns: The bacterial species name with the total number of UPCs indicated in brackets at the start of the description. Purple, High GC Gram+ bacteria; Blue, Firmicutes; Yellow, a-proteobacteria; Light Brown, b-proteobacteria; Dark Brown, e-proteobacteria; Green, g-proteobacteria (non-enterobacteria); Red, g-proteobacteria (enterobacteria); Black, others (CFB). Rows: The motif regular expression with the total number of incidences in UPCs across all 60 organisms indicated in brackets at the start of the description. Color scale: The logarithm of the normalized N_UPC returned from the SLiMSearch results.

We also looked at the distribution of known motifs (see Tables 1, 3A) across species (Figure 3). While some effector motifs (see second section of Table 1) show a wide phylogenetic distribution, others are restricted to only a few species, such as the G.LR… T motif involved in Rho GAP function. The nuclear localization signals (at the bottom of Figure 3) show a relatively restricted distribution. The WEK[IM]..FF late endocytic compartment localization motif is restricted to the genus Salmonella. While the ubiquitin ligase motifs L….TC and C.D are found in more than 71 and 199 instances respectively across the dataset whereas a number of species lack one or both of these motifs. The two SH3 binding motifs [RKY]..P..P and P..P.[KR] also show a restricted distribution. Similarly, the two PDZ binding motifs,…[ST].[ACVILF]$ and … [VLIFY].[ACVILF]$ show a restricted distribution. Bacterial effector proteins may under certain circumstances be under negative selection to avoid motifs that bind to common domains in the host such as PDZ and SH3 domains.

**Figure 4:**
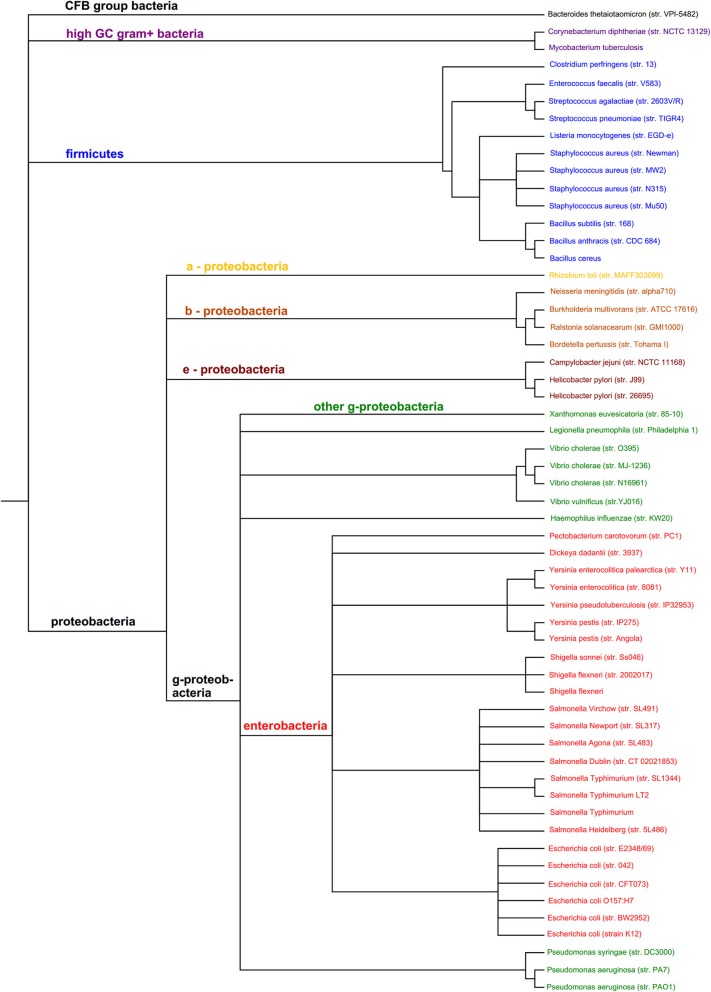
**Phylogenetic tree of the 60 organisms used to assess the distribution of prokaryotic protein motifs in Figures 2, 3**. Purple, High GC Gram+ bacteria; Blue, Firmicutes; Yellow, a-proteobacteria; Light Brown, b-proteobacteria; Dark Brown, e-proteobacteria; Green, g-proteobacteria (non-enterobacteria); Red, g-proteobacteria (enterobacteria); Black, others (CFB).

It can be seen that strains of a species often have very similar motif distributions (Figures 2–4). There is a weak but not convincing trend (Figure 3) for the known motif distribution among effector proteins of the Firmicutes (Blue) to group together, relative the gamma-proteobacteria (Red and Green). While the Group 2 Bacillus species, anthracis and cereus, cluster together (Figure 3), many sets of closely related species (Figure 4) do not show particularly close relationships in terms of motif distribution. This may result from two factors: firstly, motifs are highly dynamic during evolution, and secondly, factors that play a role in pathogenicity also evolve very fast. It is also difficult to compare rare vs. common motifs, since rare ones may be missed simply because of variation among proteins in the definition of effector proteins, while common motifs may be dominated by false positives that obscure the biologically relevant signals.

## Discussion

We believe that SLiMs are one potential class of new antimicrobial substances for the development of antimicrobial peptides and drugs. While they may lack the potency of antimicrobial peptides that damage the bacterial membrane, they may have other benefits. In particular, those that mimic peptide components of uniquely prokaryotic motifs are likely to have less off-target effects. The value of developing such therapeutic approaches depends on the range of species likely to be affected by the peptide therapeutic. While targeting eukaryotic peptides mimicked by prokaryote effector proteins provides a potential line of attack, the evolutionary plasticity of such motifs in both bacteria (Figure 3) and in hosts (Neduva and Russell, 2005) suggest that bacteria can rapidly evolve alternative effector strategies to replace one targeted host component with another. Nevertheless, where such drugs are developed for other indications in treating non-infectious disease, they may also have an impact on bacterial pathogenesis and would certainly be worth investigating. This problem of evolutionary evasion by pathogens is also relevant, however, to many adhesion motifs. In order for peptide therapeutics to be more robust in the face of rapid evolution of pathogen resistance, they may need to target fundamental components of bacterial biology. Targeting aspects of the central machinery of bacterial Type IV secretion systems may be a good compromise between targeting a component that is central to pathogenicity, while not affecting the biology of advantageous bacteria in the host. In this respect, the G[AG].$ motif identified in this study is a potential candidate worthy of further investigation. Some clues as to the function of this motif may be provided by the pattern of evolution. Presumably this motif has evolved in multiple components of the Type IV secretion system because of a selection pressure for these proteins to interact with some common factor. Identifying the common interaction partners of these proteins may help in pinpointing its potential functional role. In targeting such pathogenicity systems, the benefit of focusing on recurrent motifs is that they may be small enough interaction surfaces to be feasibly targeted by peptidomimetics, and important enough that it is difficult for the bacterial system to evolve resistance (Baron and Coombes, 2007; Paschos et al., 2011).

The shortlist of predicted motifs that we have generated provides a resource for researchers interested in the mechanisms of action of virulence factor proteins across a diverse range of bacterial species. The limitations of the list are well-illustrated by the fact that the motif discovery failed to rediscover the many mimicked eukaryotic motifs. This reflects not only the fact that some motifs have not evolved multiple times in unrelated proteins, but also the limitations in the datasets provided to the SLiMFinder approach. Ideally, datasets should have less than a 100 proteins which have clearly identified similar functions. The challenge is to group proteins according to function efficiently, since the annotation of protein function is highly variable, and frequently relies on computational predictions arising from homology rather than from direct experimentation. The bigger challenge is how to test and manipulate these motifs to provide insights into the mechanisms of action and to determine potential means of interrupting pathogenic processes. While mutagenesis studies can identify the key features of motif function, targeting of a motif may also be progressed by experimental use of bioactive peptides. However, identification of more potent peptidomimetic compounds that resemble such motifs will ideally need 3D models of the peptide regions in complex with their target interactors.

What, then, is the contribution that computational screening of novel motifs may play in the discovery of novel antimicrobial peptides? Firstly, it clearly will not identify all known motifs, since patterns of recurrent evolution or of strong sequence conservation are not seen for all antimicrobial peptides. Computational screens will also have some “false positives” in two senses: firstly, statistical false positives where the motif arose simply by chance; and secondly, biological false positives where the motif that functions effectively within its biological context of a larger protein and that protein's complexes, but it will not function as a stand-alone synthetic peptide. This could reflect a lack of strong affinity for its targets or it could reflect an inability to be delivered to the appropriate context in the first place. Nevertheless, computational screens have the advantage that they can be performed on high throughput sequencing of organisms about which little else is known and for which biological screening by mutagenesis is painstaking or impossible. The advantage of computational prioritization is that it identifies a subset of peptides which are enriched for biologically active peptides. Clearly, the strategy we adopted here is only detecting a small fraction of known motifs, in part because of the stringent correction for statistical mismatches that could be false positives, but also because many motifs do not recur in known unrelated proteins that fall into the same functional class. Discovery for bioactive peptides could follow other strategies, including searches for evolutionary conservation (Davey et al., 2012a). However, pathogenicity factors frequently evolve rapidly, and so conservation may not be an effective signal. Bioactivity predictors based on biophysical properties within the peptide sequences are an alternative strategy (Dosztanyi et al., 2009; Thomas et al., 2010; Mooney et al., 2012, 2013). These have the disadvantage that there is no straightforward statistical approach available to determine likely false discovery rates, but are very valuable in prioritizing a list of peptides for further experimental characterization. Other computational approaches focus more on particular classes of antimicrobial peptides with a strong therapeutic potential, including ribosomal and non-ribosomal cyclic peptides (Prieto et al., 2012; Kedarisetti et al., 2014). While their computational screening methods have the benefit that they focus more strongly on peptides in classes of known therapeutic benefit, we believe that the computational screening approach we identified here complements their approaches, and widens the diversity of peptides for experimental investigation and validation.

### Conflict of interest statement

The authors declare that the research was conducted in the absence of any commercial or financial relationships that could be construed as a potential conflict of interest.

## Supplementary material

Table S1 **Protein sequence identifiers for each functional category downloaded from the MvirDB**.
